# Verbal autopsy analysis of maternal mortality in Bong County, Liberia: a retrospective mixed methods study

**DOI:** 10.1136/bmjoq-2022-002147

**Published:** 2023-04-19

**Authors:** Haeun Lee, Joseph Perosky, Madison Horton, Christopher Reynolds, Aloysius Nyanplu, Jody R Lori

**Affiliations:** 1 Center for Global Health Equity, University of Michigan, Ann Arbor, Michigan, USA; 2 Obstetrics and Gynecology, Michigan State Universtiy, Lansing, Michigan, USA; 3 School of Nursing, Columbia University, New York, New York, USA; 4 Michigan Medicine, University of Michigan, Ann Arbor, Michigan, USA; 5 Bong County Health Team, Suakoko, Liberia; 6 School of Nursing, University of Michigan, Ann Arbor, Michigan, USA

**Keywords:** Continuous quality improvement, Quality improvement, Obstetrics and gynecology

## Abstract

**Background:**

While the medical contributors to maternal mortality are well known, the contextual contributors are less known and understudied. Liberia has one of the highest maternal mortality rates in sub-Saharan Africa, with recent increases in maternal deaths in rural Bong County. The purpose of this study was to better classify the contextual factors leading up to maternal deaths and to develop a list of recommendations to prevent future similar deaths.

**Methods:**

A retrospective mixed method study was conducted examining 35 maternal deaths in Bong County, Liberia using verbal autopsy reports from 2019. An interdisciplinary death audit team reviewed and analysed the maternal deaths to determine the contextual causes of the maternal death.

**Findings:**

This study identified three contextual causes: limited resources (materials, transportation, facility, staff), inadequate skills and knowledge (staff, community, family, patient), and ineffective communication (between providers, between health facilities and hospitals, and between provider and patient/family). Of these, inadequate patient education (54.28%), inadequate staff education and training (51.42%), ineffective communication between health facilities and hospitals (31.42%), and inadequate materials (28.57%) were most frequently mentioned.

**Conclusion:**

Maternal mortality remains an issue in Bong County, Liberia, due to addressable contextual causes. Interventions to ameliorate these preventable deaths include ensuring availability of resources and transportation through improved supply chain and health system accountability. Recurring training should be provided to healthcare workers which involves husbands, families and communities. Innovative means for providers and facilities to communicate clearly and consistently should also be prioritised to prevent future maternal deaths in Bong County, Liberia.

**WHAT IS ALREADY KNOWN ON THIS TOPIC**

- While the medical contributors to maternal mortality in sub-Saharan Africa are well known, the contextual contributors are less known and understudied.

**WHAT THIS STUDY ADDS**

- A retrospective mixed methods study using verbal autopsy forms found that limited resources, inadequate skills and ineffective communications were the main contextual causes of the recently increased maternal deaths in Bong County, Liberia.

**HOW THIS STUDY MIGHT AFFECT RESEARCH, PRACTICE OR POLICY**

- Availability of resources and transportation, expanding and equipping healthcare workforce, and improving communications between women, women’s family, within and between healthcare facilities need to be prioritised to prevent future similar maternal deaths.

## Background

Maternal mortality is a global health problem, with the majority of the maternal deaths occurring in low-income and middle-income countries (LMICs).^1^ Liberia has one of the highest maternal mortality ratios (MMR) among LMICs with 742 deaths per 100 000 live births.^2^ While Liberia was making significant progress towards reducing maternal mortality, a 14-year long civil war (1989–2003) and the Ebola epidemic (2014–2016) severely challenged the country’s infrastructure for maternal care.^3^ Prior to the civil wars, Liberia had 293 functioning public health facilities. However, by the end of the civil unrest in 2003, this number dropped to 51.^4^ Many healthcare workers also fled the country, leaving only 30 physicians to serve a population of 3 million.^4^

After the devastating effects of the civil war on the health system, Liberia’s government, with assistance from donors and international non-governmental organisations, launched the national health plan in 2007 to improve healthcare services.^5^ Maternal, newborn, child health, as well as reproductive, and adolescent health were key components of the plan.^5^ Furthermore, the National Health Policy and Plan 2011–2021 aimed to increase the number of high performing facilities and institutes and strengthen the workforce to be people-centred, gender-sensitive and service oriented.^6^ With such prioritised efforts, the number of health facilities grew from 51 to 727 between 2003 and 2016.^6^

As Liberia was beginning to recover from the civil wars, its health system was overwhelmed by the 2014–2016 Ebola outbreak. Many health facilities were closed due to limited supplies and resources, and utilisation of maternal health services significantly declined. For example, approximately 16 000 antenatal care visits were recorded in January 2012, which dropped to 4000 by October 2014.^7^ Furthermore, facility-based deliveries decreased by 30% or more.^6^ Experts reported people’s fear of contracting Ebola at the health facilities and the lack of trust in the health system as explanation for such underutilisation of services.^7^ As an alternative, many women sought services from traditional birth attendants, or received no services at all, which increased pregnancy and childbirth related deaths and injuries.^7^ Confounded by socioeconomic and cultural factors, maternal mortality is highest for unsupervised deliveries occurring in rural and poor communities, and the deaths occurring outside of health facilities are often not properly examined nor recorded.^8^ Without proper records, the causes and contributing factors of death cannot be captured to prevent future deaths and injuries.^8^

Bong County is the third most populous county in Liberia with increased maternal deaths in recent years. In 2014 and 2015, there were fewer than 10 maternal deaths each year. However, between 2016 and 2019, maternal deaths increased to approximately 35 each year. To better understand the various contributing factors and sequential events of the maternal deaths, the Bong County’s Community Health Teams (CHTs) conducted verbal autopsies (VAs) on all maternal deaths that occurred in 2019. VA is a method widely used in settings where deaths occur without medical supervision.^9^ To understand the factors contributing to the high maternal mortality in Bong County, Liberia, a retrospective mixed method analysis was conducted using VA reports.

## Methods

This descriptive retrospective mixed methods analysis examined VAs of 35 maternal deaths that occurred in Bong County, Liberia in 2019.

### Setting

Liberia is a country located in western Africa with a population of 5.19 million. With an MMR of 742 deaths per 100 000 live births, Liberia is approximately 8.2 years behind its national target to reach 496 deaths per 100 000 by 2021.^10^ In 2016, there were 727 health facilities including hospitals, clinics and health centres in Liberia.^6^ It has less than 1.5 skilled birth attendants per 1000 people, which is below the minimum threshold of 2.3 skilled providers required to ensure women have access to a skilled provider at birth for 80% of the population.^7^ Bong County is in the north-central portion of Liberia and consists of 12 districts.^11^ Of the 15 counties in Liberia, Bong County is the third most populated county with a population of 328 919.^11^ There are currently 3 hospitals and 45 rural health facilities in Bong County.^12^

### Instrument

The VA reports were collected using the Maternal Death Investigation and Reporting Form (MDIRF). In Liberia, Maternal and Newborn Death Verbal Audits were not routinely collected until 2016. There was high variability between CHTs regarding the quality and content of the audit reports prior to this time. In 2016, at the Liberia Maternal and Newborn Health Conference, the Ministry of Health decided to implement mandatory countrywide audit reporting forms for maternal and newborn deaths which occur in any setting, including both health facility and community-based deaths.

To this means, the MDIRF was created (online supplemental appendix 1). When a death is reported, a team of 1–2 members of the reproductive health division of the CHT travel out to the location of death or in the case of a facility death to the home community of the deceased woman. The MDIRF form consists of both closed ended and open-ended questions with six main sections: (1) reporting information (eg, date of death and investigation), (2) deceased woman’s demographics information (eg, age, marital status, education level), (3) reproductive health history (eg, gravida/parity, antenatal care visits), (4) maternal death circumstances (eg, stage of pregnancy at the time of death, delivery location, referral status, mode of transportation), (5) reported cause of death (primary, secondary cause of death recorded by healthcare provider present at death) and (6) overall interpretation of the collected data and recommendations. Sections 1–5 consist of closed-ended questions and are collected from the deceased women’s family members and/or healthcare provider(s). Section 6 uses open-ended questions and is completed by the CHT member who collected the data. All data are first collected on paper copies and transferred to an Excel sheet or word document. These tools aim to generate information on maternal and newborn deaths to inform future decision-making and interventions to reduce preventable mortality.^7^

10.1136/bmjoq-2022-002147.supp1 Supplementary data

### Data analysis

An interdisciplinary death audit team was developed consisting of researchers from the University of Michigan and representatives from the CHT, analysed the data together to include diverse investigators who are proficient in health sciences, systems thinking and the local contexts.^13^ All identifiable information about the deceased women was removed before analysis. The quantitative data from the first five sections of the VA were analysed using Stata 17 (StataCorp). The primary causes of deaths were coded by three independent medically trained researchers after reviewing the first five sections. Next, the researchers compared the codes and discussed until unanimous agreement was achieved. The cause of deaths was recoded by the researchers for data quality and consistency after an in-depth review of the VA data.

Two of the three researchers also reviewed the entire VA data including qualitative pieces, paying special attention to the sequenced maternal death circumstances from the moment a woman decided to seek help until death. The coding was inductive, where each of the investigators independently analysed and developed codes from the VA forms then reviewed, compared, overlapped and categorised the causal factors. Together, the researchers developed a codebook, which was then used to hand-code the data independently. The coded data were then compared and discussed until all codes reached consensus among study investigators. After all the quantitative and qualitative data were analysed, the death audit team collectively developed a list of recommendations on how to address the contextual causes (main themes) and contributing factors (subthemes) to prevent similar deaths.

## Results

### Demographics

A total of 35 VA reports were collected from deceased women’s family and healthcare providers that cared for women in Bong County, Liberia. Table 1 presents the demographic data of the deceased women. Twelve (34.2%) of the 35 deceased women were between 31 and 35 years old and 10 (28.5%) of the women were between 16 and 20 years old. The majority of the women were married or cohabiting (71.4%) and the half of them were farmers (51.4%). Sixteen (45.7%) women had more than 5 pregnancies and 11 (31.4%) women had more than 5 live births. Among the risk factors, infectious diseases (eg, malaria/fever/syphilis/hepatitis) were most common (51.4%). Obstetric history included 71.4% of the women having a history of a stillbirths, 31.4% had a previous abortion and 14.2% had a previous caesarean section. More than half of the women (60.0%) received antenatal care for the current pregnancy. All percentages for the tables took the missing observations into consideration.

**Table 1:** Demographic characteristics and reproductive history

Characteristics	N (%)
Total	35 (100.00)
Age	
16–20	10 (28.57)
21–25	4 (11.43)
26–30	3 (8.57)
31–35	12 (34.29)
36–40	2 (5.71)
>41	4 (11.43)
Marital status	
Married/cohabiting	25 (71.43)
Widowed/divorced	–
Single	4 (11.43)
Occupation	
Farmer	18 (51.43)
Businesswoman	3 (8.57)
Student	3 (8.57)
Housewife	4 (11.43)
Gravida	
1	7 (20.00)
2	2 (5.71)
3	3 (8.57)
4	6 (17.14)
≥5	16 (45.71)
Parity	
0	6 (17.14)
1	3 (8.57)
2	3 (8.57)
3	3 (8.57)
4	7 (20.00)
≥5	11 (31.43)
Risk factors*	
Hypertension/eclampsia/ pre-eclampsia	2 (5.71)
Malaria/fever/syphilis /hepatitis	18 (51.42)
Anaemia	7 (20.00)
Abnormal lie/mal presentation	3 (8.57)
Bleeding	10 (28.57)
Others	6 (17.14)
HIV	1 (2.86)
Sickle cell anaemia	0 (0.00)
Previous stillbirth	
No	10 (28.57)
Yes	25 (71.43)
Previous abortion	
No	24 (68.57)
Yes	11 (31.43)
Previous C-section	
No	28 (80.00)
Yes	5 (14.29)
Received ANC for this pregnancy	
No	8 (22.86)
Yes	21 (60.00)
Don’t know	5 (14.29)

All percentages were calculated with missing observations in consideration.

*Questions asked to mark all that apply.

ANC, antenatal care.

### Maternal death circumstances

All women (n=35) travelled to a rural health facility, staffed by a nurse or a midwife, to seek care. Table 2 presents the maternal death circumstances leading up to death. When travelling from home to a health facility, 14 (40.0%) women took a commercial vehicle, 8 (22.8%) took an ambulance and 6 (17.1%) walked. Travel time to the nearest health facility was 30 min or less for 31.4% of the women. Twenty-one (60.0%) women were referred from a rural health facility to a district hospital capable of providing more comprehensive emergency obstetric care such as caesarean section and blood transfusion. One-third of these women took an ambulance and another one-third used a commercial vehicle to get to the hospital. For women referred from a rural health facility, travel time took less than 30 min for 5 (23.8%) women, between 31 min to an hour for 3 (14.28%) of the women and more than an hour for 8 (38.08%) women to arrive at the hospital.

**Table 2:** Sequential event leading up to maternal death

Maternal death circumstances	N (%)
Mode of transportation from home to health facility	
Motorcycle	4 (11.43)
By foot	6 (17.14)
Hammock	2 (5.71)
Ambulance	8 (22.86)
Commercial vehicle	14 (40.00)
Private vehicle	1 (2.86)
Time taken to travel from home to nearest health facility	
≤30 min	11 (31.43)
31 min to 1 hour	2 (5.71)
1–1.5 hours	2 (5.71)
1.5–2 hours	2 (5.71)
>2 hours	1 (2.86)
How long until receive care at health facility	
≤5 min	18 (51.43)
6–15 min	4 (11.43)
16–30 min	5 (14.29)
≥31	2 (5.71)
Treatment received at health facility*	
Intravenous/plasma/blood	28 (80.00)
Antibiotics/uterotonics/antiseizure/calcium gluconate	28 (80.00)
Received herbal treatment prior to admission	
No	5 (14.29)
Yes	3 (8.57)
Don’t know	23 (65.71)
Women referred to hospital	
No	11 (31.43)
Yes	21 (60.00)
Delivery monitored with partograph	
Yes	14 (40.00)
No	12 (34.29)
Mode of transportation from health facility to hospital†	
Motorcycle	1 (4.76)
Hammock	3 (14.28)
Ambulance	7 (33.33)
Commercial vehicle	7 (33.33)
Private vehicle	1 (4.76)
Time travel from health facility to hospital†	
≤30 min	5 (23.80)
31 min to 1hour	3 (14.28)
1–1.5 hours	3 (14.28)
1.5–2 hours	2 (9.52)
≥2 hours	3 (14.28)
Type of skilled provider present at birth	
Midwife	18 (51.43)
Physician’s assistant	1 (2.86)
Medical doctor	4 (11.43)
Others	2 (5.71)
Time of death	
Antepartum	11 (31.43)
Intrapartum	2 (5.71)
24 hours post partum	14 (40.00)
Up to 42 days post partum	5 (14.29)
Type of delivery‡	
Assisted vaginal delivery	1 (4.16)
Induced vaginal delivery	3 (12.5)
Spontaneous vaginal delivery	9 (37.50)
C-section	8 (33.33)
Place of delivery	
Health facility	17 (48.57)
Outside of health facility	6 (17.14)
Place of death	
Home	2 (5.71)
Health facility/hospital	30 (85.71)
On the road	3 (8.57)
When death occurred	
Rainy season (May–October)	22 (62.86)
Dry season (November–April)	12 (34.29)
Baby status	
Alive and healthy	12 (34.29)
Alive but critical	2 (5.71)
Fresh stillbirth	4 (11.43)
Macerated stillbirth	3 (8.57)
Primary medical cause of maternal death	
Haemorrhage	11 (31.43)
Sepsis	8 (22.86)
Pre-eclampsia/eclampsia	1 (2.86)
Ruptured uterus	2 (5.71)
Abortion	2 (5.71)
Indirect causes§	11 (31.43)

All percentages were calculated with missing observations in consideration.

*Questions asked to mark all that apply.

†Percentages calculated out of 21 women who were referred to hospitals.

‡Percentages calculated out of 24 potential women who died intrapartum or post partum.

§Indirect causes of maternal deaths include malaria, anaemia, embolism, etc.

Eleven (31%) women died prior to delivery, 2 (5.7%) during delivery, 14 women (40.0%) within 24 hours of post partum and 5 (14.2%) within 42 days of post partum. Among women who died during delivery or postpartum period, 37.5% women had a spontaneous vaginal delivery, 33.33% had a caesarean section, 12.5% had induced vaginal delivery and 4.1% had assisted vaginal delivery. Only half of the women (48.5%) delivered at a health facility, but 71% of the women with a skilled provider present during delivery. This finding indicates that 22.5% of the women had a skilled provider outside of a health facility. The majority of the women died at a rural health facility or a hospital (85.7%). More women died during rainy season (62.8%), which is from May to October. One-third of the babies were alive and healthy, two were alive but in critical condition and another seven were stillbirths. Primary medical causes of maternal death were haemorrhage (50.0%), sepsis (32.3%), indirect causes (11.7%), hypertension (2.9%) and embolism (2.9%).

### Contextual causes and contributing factors of maternal deaths

Table 3 shows the tabulated contextual causes and the contributing factors of the maternal deaths. There are three contextual causes identified: (1) limited resources, (2) inadequate skills and knowledge and (3) ineffective communication.

**Table 3:** Contextual causes of maternal deaths

Contextual causes	Total deaths	Contextual causes of maternal death N (%)	
		Directly mentioned	Indirectly inferred
	35	24	11
Limited resources			
Materials	10 (28.57)	6 (25.00)	4 (36.36)
Transportation	7 (20.00)	6 (25.00)	1 (9.09)
Facilities	2 (5.71)	2 (8.33)	–
Staff	4 (11.42)	3 (12.50)	1 (9.09)
Inadequate skills and knowledge			
Staff education and training	18 (51.42)	12 (50.00)	6 (54.54)
Community and family education	9 (25.71)	4 (16.66)	5 (45.45)
Patient education	19 (54.28)	11 (45.83)	8 (72.72)
Ineffective communication			
Between providers	6 (17.14)	6 (25.00)	–
Between health facility and hospital	11 (31.42)	8 (33.33)	3 (27.27)
Between providers and patient/family	4 (11.42)	4 (16.66)	–

#### Limited resources

Materials, transportation, facility and staff were identified as contributing factors under limited resources. Limited materials (28.5%) were the most frequently identified, closely followed by transportation (20.0%). Limited material resources included oxygen, medication, intravenous fluids, blood products and equipment including partographs at the facilities. In one instance, relatives were asked by providers to prepare necessary materials for delivery, including donating their own blood due to hospital shortage. When the relatives refused to donate blood and were not able to prepare the requested materials on time, the woman died. Limited transportation means, including ambulances were also significant contributors to maternal deaths. One of the deceased women’s providers stated, ‘The patient started bleeding and the ambulance was called at 10:50am. We were told that the ambulance was down and another ambulance at a close by hospital did not have fuel. At 12:10pm patient was taken in a commercial vehicle but died on route.’ Similarly, there was often only one ambulance available, causing delays for the next woman in need of an emergency transfer. Family members would then try to find commercial transportation which often took a significant amount of time and family resources.

Limited facilities such as operating rooms and maternity waiting homes, lodging facilities for women who live far away from health facilities and/or have high-risk pregnancy to await delivery, contributed to delay in receiving care. One of the deceased women had to wait more than 12 hours to receive a caesarean section because there was no operating room available. In other instances, there were no maternity waiting homes for the women living far distances from a health facility to stay and await delivery. They were forced to travel long distances while in labour in order to access services. The lack of adequate numbers of healthcare providers as well as the types of providers available was also mentioned. There were insufficient lab technicians to perform timely and necessary labs for prompt diagnosis and care. Already overburdened providers struggled to provide care quickly and effectively for all patients or to properly supervise and mentor newer providers.

#### Inadequate skills and knowledge

Inadequate skills and knowledge included staff education and training (51.4%), patient education (54.2%) and community and family education (25.7%). Multiple VA reports showed that healthcare providers misdiagnosed or simply did not identify the complications that led to the adverse outcomes. A deceased women’s sister commented, ‘she stayed in the ER for two days before she was sent to the OB ward. The doctor ordered oxygen but there was no oxygen for her.’ Such incidence shows both limited obstetric triage and inadequate staff training. One of the providers mentioned ‘the death could have been prevented if the complication was identified earlier… the intrauterine fetal death was not identified, and the patient was kept in the maternity waiting home for two weeks. The fetus was macerated, and the patient became septic when she was referred to the hospital.’ In another instance, a friend of a deceased women said that the patient visited the clinic twice, but she was sent home, where she remained for two days.

Limited patient and community and family education were also frequently mentioned (25.7%). The VA analysis identified insufficient education and emphasised around the importance of early and frequent antenatal care, available services, recognition of danger signs and debunking traditional medicines that are harmful to patients at the community level. Many of the deceased women had symptoms of danger signs such as vaginal bleeding, convulsion, fever, abdominal pain and fast breathing which were not identified by the patients or their families until these symptoms became severe. While these women can and should receive education on the importance of facility delivery and danger signs during antenatal care visits, the family and community members who are often in very close proximity with the women also need to be included in training to recognise and bring women to a healthcare facility or to support her when she needs emergent care. One instance detailed a severely sick woman requiring her to be transferred to the hospital from the rural health facility. When the parents and the relatives were informed, they refused. Later she went home with the family. Her symptoms worsened and she died on route being transferred to the hospital. In other instance, a family member reported they realised something was wrong but did not know how to properly seek help. Both deaths could have been prevented if the family and the community members were also aware of the danger signs and knew the appropriate next steps to seeking care.

#### Ineffective communication

Ineffective communications between providers, between health facility and hospital, and between providers and patient/family were also identified as contributors to maternal deaths. Between providers, information and updates regarding the patient care were not being communicated effectively. In one instance, a patient spent 23 hours in the emergency room (ER) before the ER nurse informed the on-call doctor. There were significant delays in communicating patient histories from the rural health facility to the hospital and between the nurses and the doctors. In many cases, the doctors would put in the order, the nurses would deliver the care, but not update the doctor about patient status after delivering care nor would the physician follow-up and tell the nurse whether he/she checked on the patient.

Patients and patient families did not receive sufficient updates regarding patient status to make collective decisions regarding patient care. In cases where patients and family members were informed, insufficient explanations regarding the potential consequences were often given. Hence, there were multiple instances where the family was told to transfer the patient to a hospital without further information on what to tell the healthcare providers once they arrived at the facility. In other instances, patients and family members would not disclose the full information to the providers. One of the providers commented, ‘the woman initially denied any form of induced abortion… about two days after admission, she admitted that she had two herbal concoction a week prior to coming to the hospital’. As such, failed communication at multiple levels contributed to maternal deaths.

Table 4 presents the contextual causes, contributing factors and means to address each of the contributing factors per death audit team recommendation.

**Table 4:** Contextual causes, contributing factors and recommendations

Contextual cause	Causal factor	Recommendations
Limited resources	Materials	Prevention of stockouts through supply chain planning, regular inventory assessment and distribution planning Improve health facility accountability to prevent material appropriation
	Transportation	Identifying resources within the community to support travel Network between hospitals with ambulance Ensure ambulances maintenance
	Facility	Increase maternity waiting homes at rural facilities Coordination for transfer of patients between facilities that are over capacity
	Staff	Management protocols to address staffing Prioritisation of assignments Examine staff mix Examine and reassign non-provider tasks done by healthcare professionals
Inadequate skills/knowledge	Staff education/training	Providing continuing education and triage skills to providers of obstetric care Development of clear up-to-date protocols for low, intermediate and high-risk patients
	Community/family education	Involving husbands, partners and families in antenatal care Proving community-wide education
	Patient education	Providing information in a way that women can acquire, understand and use health messages to promote and maintain health
Limited/ineffective communication	Between providers	Document sufficient details in the medical record and in referral forms
	Between rural health facility and hospital	Communicate through text messages or other technology before, during and after transfer of women
	Between providers and patient/family	Improving health literacy Improve trust

## Discussion

While the top causes of maternal mortality worldwide are most often listed as major medical complications, including haemorrhage, sepsis, hypertension and unsafe abortion, this study enhances our understanding of the contextual causes that contribute to maternal outcomes for women presenting with these complications.^1^ This analysis of 35 cases of a maternal death confirmed that limited resources, inadequate skills and limited/ineffective communication were the main contextual, non-medical causes of maternal mortality. It presents a comprehensive understanding of the contributing organisational, sociotechnical and structural factors that impact obstetrical outcomes. Similarly, it provides us with data on how health system structures, providers and community factors contribute to the overall quality of care.^14^

Limited materials at the healthcare facility, including oxygen, intravenous fluids, medications and equipment, were most often cited under limited resources. Adequate supplies are fundamental to providing quality care to obstetric patients. Supply chain planning and increased accountability in the healthcare system to assure the availability of resources is crucial to improve maternal outcomes. Similar to our findings, another study of 30 maternal deaths in Indonesia found that lack of equipment and supplies contributed to 23% of maternal deaths.^14^

Limited transportation and availability of maternity waiting homes for women who live far distances from a health facility were also identified as contributing factors. More women took commercial vehicle compared with ambulances when travelling form home to rural health facility. One-third took commercial vehicle and another one-third took ambulances from the rural health facility to hospitals, indicating the scarcity of ambulances and the burden put on the patients and their family to secure transportation in case of emergency. Furthermore, 22.5% of the women delivered outside of health facility with a healthcare provider, indicating that the patients could not get to the facility on time, requiring the providers to travel to provide care. Stronger emphasis on birth planning and community engagement to identify potential sources of transportation or short-term loans to help pay for transportation should a woman from a rural area need to be transferred could help address this issue. In a study conducted in Botswana, half of 82 maternal deaths identified some barrier to accessing health services.^15^

Inadequate skills and knowledge were pervasive from the community level to hospital staff. Rural healthcare facility and hospital staff need continuing education on assessment and triage skills. Our data showed that haemorrhage and sepsis together contributed to more than 80% of the medical causes of maternal deaths. Both of which could have been addressed through clear protocols, competent skills and timely provision of care. Hence, regular quality improvement processes including team drills can contribute to problem-solving and keep staff updated on evidence-based practice.^15^

Inclusion of husbands or partners in antenatal care has shown a positive associate between women receiving antenatal care from a skilled provider, delivering at a health facility and seeking care from for obstetric complications from a healthcare provider.^16 17^ Men often have control of the resources needed for transportation to a higher-level facility and need to be better involved in the pregnancy and delivery process. Including men and family members in knowledge acquisition and planning for a birth using culturally appropriate methods and materials could fill this gap. How women and families obtain and use health information to make decisions is complex and is influenced by the social determinants of health.^17^

Finally, effective communication must be strengthened at all levels, including between providers at the same facility, between rural health facilities and district hospitals, and between providers and patients/families. There are numerous possibilities available for electronic communication. Low-cost mobile devices have the potential to revolutionise access to seamless healthcare across the continuum in low-resource settings. Mobile instant messaging applications such as WhatsApp have seen a global proliferation over the past several years. There are currently 1.5 billion users of the free WhatsApp platform in 180 countries.^18^

Electronic communication via messaging applications can not only enhance communication between providers and health facilities but also simplify and clarify documentation process. Clear and complete documentation is necessary to keep track of patient progress and plan of care. Hence, the use of messaging application can be an innovative way to keep all the providers on the same page regarding each patient’s status. Follow-up on all maternal deaths must be ensured to develop recommended actions to prevent future deaths.

### Limitations

This study has several limitations. First, it is subject to recall bias since it is a retrospective analysis. Some interviews with family members, community members and healthcare providers took place months after the incident. Second, while the CHT interviewed the relevant stakeholders for each death, they may have missed important stakeholders that could have provided additional information. Third, because this study is a case series study specific to Bong County, Liberia, generalisation may be limited. Despite these limitations, this study provides critical insights regarding the contextual causes contributing to maternal deaths.

## Conclusion

While the medical causes of maternal mortality are well known, the contextual causes are less understood and understudied. This retrospective mixed method study conducted an analysis on 35 maternal death using VA forms. It found that limited resources, inadequate skills and ineffective communications were the main contextual causes of recently increased maternal deaths in Bong County, Liberia. Improved supply chain, healthcare system accountability and community engagement are critical to assure the availability of resources and transportation needed to address reproductive emergencies. Liberia must also prioritise expanding healthcare workforce and better equip healthcare providers’ skills, knowledge, and interfacility and intrafacility communication through continued education and team drills. Widespread education and inclusion of the women’s husbands, family and community are also critical. Finally, innovative means such as electronic messaging applications also need to be incorporated for effective communication and documentation.

### Patient and public involvement

- When and how were patients/public first involved in the research?
  Bong County Health Team (BCHT) first brought up the idea to conduct a verbal on the deceased women to better understand the contextual causes of maternal deaths.
- How were the research question(s) developed and informed by their priorities, experience and preferences?
  The overall research question was brought up by the CHT, asking academic partners to assist with data analysis and interpretation. Data collection process heavily involved the CHT and the entire study was conducted in a collaborative process.
- How were patients/public involved in design, choice of outcome measures and recruitment for the study?
  The design, outcome measures, tools used and the recruitment (of healthcare providers and deceased women’s family) were pre-established by the Liberia government and the CHT, as mentioned in the Methods section of the manuscript.
- How were (or will) patients/public be involved in choosing the methods and agreeing plans for dissemination of the study results to participants and linked communities?
  We have discussed and agreed on how to disseminate the finding in academic settings (journals, academic partners in charge) and in local settings (local communities and administrative leaderships, BCHT in charge).

## Data Availability

Data are available on reasonable request.

## References

[1] Say L, Chou D, Gemmill A, et al. Global causes of maternal death: a who systematic analysis. Lancet Glob Health 2014;2:e323–33. 10.1016/S2214-109X(14)70227-X 25103301

[2] Ministry of Health, R. of U. Liberia demographic and survey, 2019-20. 2019.

[3] Yaya S, Uthman OA, Bishwajit G, et al. Maternal health care service utilization in post-war liberia: analysis of nationally representative cross-sectional household surveys 11 medical and health sciences 1117 public health and health services. BMC Public Health 2019;19:1–12. 10.1186/s12889-018-6365-x 30621669 PMC6323818

[4] Kruk ME, Rockers PC, Williams EH, et al. Availability of essential health services in post-conflict liberia. Bull World Health Organ 2010;88:527–34. 10.2471/BLT.09.071068 20616972 PMC2897988

[5] Ministry of health and social welfare. In: National Health Policy, National Health Plan. 2011: 2007–11.

[6] Lavelah YN. Understanding women experiences of seeking and receiving maternity services in montserrado and bong counties, liberia: an in-depth qualitative study. Harvard University, 2020.

[7] Liberia, G. of. Investment plan for building a resilient health system in liberia. Monrovia, Ministry of Health 2015;12:1–61.

[8] D’Ambruoso L, Byass P, Qomariyah SN, et al. A lost cause? extending verbal autopsy to investigate biomedical and socio-cultural causes of maternal death in Burkina Faso and Indonesia. Soc Sci Med 2010;71:1728–38. 10.1016/j.socscimed.2010.05.023 20646807

[9] Soleman N, Chandramohan D, Shibuya K. Verbal autopsy: current practices and challenges. Bull World Health Organ 2006;84:239–45. 10.2471/blt.05.027003 16583084 PMC2627297

[10] Laaser U, Broniatowski R, Byepu S, et al. Delays in achieving maternal, newborn, and child health targets for 2021 and 2030 in liberia. Front Public Health 2019;7:386.:386. 10.3389/fpubh.2019.00386 31921750 PMC6923216

[11] Ministry of Internal Affiars, R. of L. Overview of bong county. 2008. Available: https://www.mia.gov.lr/1content.php?sub=199&related=40&third=199&pg=sp

[12] LISGIS), L. I. of S. and G.-I. S. Liberia population and housing census 2021. 2021.

[13] Peerally MF, Carr S, Waring J, et al. The problem with root cause analysis. BMJ Qual Saf 2017;26:417–22. 10.1136/bmjqs-2016-005511 PMC5530340 27340202

[14] Mahmood MA, Mufidah I, Scroggs S, et al. Root-cause analysis of persistently high maternal mortality in a rural district of indonesia: role of clinical care quality and health services organizational factors. Biomed Res Int 2018;2018:3673265. 10.1155/2018/3673265 29682538 PMC5842724

[15] Madzimbamuto FD, Ray SC, Mogobe KD, et al. A root-cause analysis of maternal deaths in botswana: towards developing A culture of patient safety and quality improvement. BMC Pregnancy Childbirth 2014;14:231. 10.1186/1471-2393-14-231 25030702 PMC4223720

[16] Rahman AE, Perkins J, Islam S, et al. Knowledge and involvement of husbands in maternal and newborn health in rural bangladesh. BMC Pregnancy Childbirth 2018;18:247. 10.1186/s12884-018-1882-2 29914410 PMC6007056

[17] Lori JR, Rominski S, Osher BF, et al. A case series study of perinatal deaths at one referral center in rural post-conflict liberia. Matern Child Health J 2014;18:45–51. 10.1007/s10995-013-1232-y 23417211 PMC3703491

[18] Iqbal M. WhatsApp revenue and usage statustics. 2022. Available: https://www.businessofapps.com/data/whatsapp-statistics/

