# Supplementary material for: Verbal autopsy analysis of maternal mortality in Bong County, Liberia: a retrospective mixed methods study
Source: BMJ Open Qual. 2023 Apr 19;12(2):e002147. doi: 10.1136/bmjoq-2022-002147 (PMC10124242; doi:10.1136/bmjoq-2022-002147)
Supplement: Supplementary data [file bmjoq-2022-002147supp001.pdf]

BMJ Publishing Group Limited (BMJ) disclaims all liability and responsibility arising from any reliance placed on this supplemental material which has been supplied by the author(s)

MINISTRY OF HEALTH

MATERNAL AND NEWBORN DEATH SURVEILLANCE AND RESPONSE GUIDELINE

LIBERIA

JULY 21, 2015

Lee H, *et al. BMJ Open Qual* 2023; 12:e002147. doi: 10.1136/bmjoq-2022-002147

Introduction
The death of a mother and new born is a tragedy that has an immense impact on the wellbeing of the family and society at large. Most causes of maternal and new born deaths in Liberia can be prevented or treated. Hence, the need for active surveillance system that documents and ensures that all deaths are investigated by trained and qualified staff and appropriate actions be taken to reduce and avoid future deaths.

A vital component of the surveillance system is the provision of information about the underlying factors contributing to maternal deaths and intervention on how they should be tackled. Response to such system should aim at eliminating preventable causes of maternal and new-born morbidity and mortality. Maternal Newborn Death Surveillance & Response (MNDSR) is a model of such a system that responds to Millennium Development Goals 4 and 5, which aim to reduce child, maternal, and new-born mortality.

What is Maternal and New Born Death Surveillance and Response (MNDSR)?
MNDSR is the on-going systematic collection, analysis, and interpretation of maternal and new born deaths data. It includes the timely dissemination of the resulting information to those who need them for action. It is essential for planning, implementation, and evaluation of public health practices.

Rationale
The rationale for MNDSR is to generate information on maternal and new-born deaths for informed decision making and appropriate actions or interventions.

Developing countries use periodic surveys that are usually expensive and time consuming as the source of information or data on maternal and new-born deaths.

MNDSR assists in computing country-owned maternal and new born deaths data. It also provides more reliable MNDSR at lower levels thereby showing where the greatest burden of mortality is located. Additionally, it provides essential information for programme, mid-year reviews and monitoring.

Goal of the MNDSR GUIDELINE:
The overall goal of the MNDSR guideline is to guide effective implementation and scale up of MNDSR in a systematic, standardized and integrated manner. **It i**s also to contribute to the reduction of maternal and new-born deaths through an improved surveillance system.

OBJECTIVES OF MNDSR:
1. Strengthen the capacity of the country to conduct effective surveillance activities: train personnel at all levels; develop and carry out plans of action; and advocate and mobilize resources.
2. Guide programme Managers in the implementation and supervision of the MNDSR
3. Facilitate standardization and harmonization of the MNDSR process at community, facility, district, county and national levels.

Lee H, *et al*. *BMJ Open Qual* 2023; 12:e002147. doi: 10.1136/bmjoq-2022-002147

4. Improve the use of information to detect changes in time in order to conduct a rapid response to maternal and new born deaths; monitor the impact of interventions: for example, declining maternal and new born deaths, planning; and management.
5. Improve the flow of surveillance information with feedback at all levels within the health system.
6. Increase involvement of clinicians in the surveillance system.
7. Emphasize community participation in reporting and response to maternal and new born deaths to prevent future deaths.
8. Trigger epidemiological investigations in detection, investigation and reporting of maternal and new born deaths.

I. Maternal Deaths

**Background**
• Hemorrhage remains the leading cause of maternal death in Liberia, and unattended births by skilled attendants are a particular risk, especially in rural areas where transport to health care facilities is nearly non-existent.<br>• Maternal mortality ratio in Liberia was estimated at 1072/100,000 live births in 2013.<br>• Review of progress towards MDG 5 indicates that Liberia is unlikely to achieve this MDG by 2015. Intensified actions and increased investments are required to improve the coverage and quality of maternal health care services at all levels in Liberia. Thus monitoring maternal deaths and addressing issues and factors contributing to these deaths are key if we are to achieve MDG 5.
**Surveillance Goal**
• **The overall goal of the MNDSR protocol is to guide an effective implementation and scale up of MNDSR in systematic, standardized and integrated manner**
**Surveillance Objectives**
• Estimate and monitor maternal mortality rates.<br>• Identify risk factors for maternal mortality to inform programs and decision makers.<br>• Investigate all maternal deaths in facilities and communities and take necessary action.
**Standard case definition**
The death of a woman while pregnant or within 42 days of the delivery or termination of the pregnancy, irrespective of the duration and site of the pregnancy, from any cause related to or aggravated by the pregnancy or its management but not from accidental or incidental causes.
**Recommended public health action**
• A case of maternal death is a trigger for action at all levels (communities, health facilities, districts counties and national).Refer to core function chart<br>• Monitor trends and respond to each alert<br>• All health care providers (professional and non-professional) should be trained on these protocols.
• **Analyze and interpret data**

Lee H, *et al*. *BMJ Open Qual* 2023; 12:e002147. doi: 10.1136/bmjoq-2022-002147

I. Maternal Deaths

Time: Graph cases to construct weekly, monthly and annual epidemic curves

Place: Plot the location of cases and analyze the distribution.

Person: Analyze the distribution of cases by age, parity and social economic status.

**Maternal Death investigation and reporting form for Health facility and community**		
This form MUST be filled by the professional health worker ***to investigate* death of a woman while pregnant or within 42 days of the delivery or termination of the pregnancy, irrespective of the duration and site of the pregnancy, from any cause related to or aggravated by the pregnancy or its management but not from accidental or incidental causes. This will be done** to identify and report primary cause(s) or key factors associated with the death with the view to take actions to prevent future maternal death.		
**Questions / Variables**		**Answers**
1	County	
2	District	
3	Name of health facility Reporting death	
4	Health facility catchment population?	
5	Place of death facility or community)	
6	Name of the death place (Community/street/quarter, clinic, health center, district hospital, referral hospital or private hospital, on the way to health facility)	
7	Date of maternal death (day/month/year) the month should be written in words for consistency at all levels	
8	**Record's unique identifier (year-Country code- County-site-maternal death rank)**	
9	Name of Deceased:	
10	Age of the deceased (in completed years)	
11	Gravida: how many times was the deceased pregnant including abortion?	
12	Parity: how many times did the deceased deliver a baby of 28 weeks or more?	
13	Marital Status: 1. Married ☐ 2. Single ☐ 3. Separated ☐ 4. Widowed ☐ 5. Living together ☐	
14	Educational Status: 1 Never been to school ☐ 2. Elementary ☐ 3. Junior High ☐ 4. Senior High ☐ 5. Higher education ☐	
15	Occupation of Deceased:	
16	Time of death (specify "During pregnancy, delivery, immediate postpartum period (1st 24hrs), or long up to 42 days after delivery ")	
17	If abortion: was it spontaneous or induced?	
18	If induced, was it medical or non-medical?	
**Maternal death history and risk factors**		

Lee H, *et al. BMJ Open Qual* 2023; 12:e002147. doi: 10.1136/bmjoq-2022-002147

**Maternal Death investigation and reporting form for Health facility and community**		
This form MUST be filled by the professional health worker ***to investigate*** **death of a woman while pregnant or within 42 days of the delivery or termination of the pregnancy, irrespective of the duration and site of the pregnancy, from any cause related to or aggravated by the pregnancy or its management but not from accidental or incidental causes. This will be done** to identify and report primary cause(s) or key factors associated with the death with the view to take actions to prevent future maternal death.		
**Questions / Variables**		**Answers**
19	Was the deceased receiving any antenatal care? (Yes /No/ unknown)	
	If yes how many times did she attend ANC?	
20	Did she have Malaria? (Yes /No/ unknown)	
21	Did she have Hypertension? (Yes /No/ unknown)	
22	Did she have Anaemia? (Yes /No/ unknown)	
23	Did she have Abnormal Lie? (Yes /No/ unknown)	
24	Did she have Diabetes	
25	Did she have convulsion/jerking (Yes /No/ unknown)	
26	Was there any bleeding (Yes /No/ unknown)	
27	Did she have Fever (Yes /No/ unknown)	
28	Did she have Hepatitis/jaundice (Yes /No/ unknown)	
29	Did she have Proteinuria	
30	Did she have Glycosuria	
31	Any other risk factor? Specify	
32	Did she undergo any Previous Caesarean Section? (Yes /No/ unknown)	
33	What was her HIV Status? (choose "HIV+; HIV-; or Unknown HIV status")	
34	Was the deceased tested for syphilis? Yes /No/ unknown	
35	If yes, what was her result? Negative / Positive	
36	Was the pregnancy a result of rape? Yes /No/ unknown	
37	Did the deceased have sickle cell anaemia ? Yes /No/ unknown	
38	**Delivery, puerperium and neonatal information**	
39	How long (hours) was the duration of labor	
40	Was the delivery monitored by pathograph? Yes / No/unknown	
41	What type of delivery was it? (choose one from "1=Vaginal non assisted delivery, 2=vaginal-assisted delivery (Vacuum), or 3=Caesarean section"	
42	What was the baby status at birth? (Alive or Stillborn/unknown)	
43	In case the baby was born alive, is he/she still alive or died within 28 days after his/her birth? (choose 1=Still alive, 2=early neonatal death (within 7 days) 3= late neonatal death (within 8-28 days) 4=died beyond 28 days of age 5=unknown)	
44	Was the birth attended to by a skilled care provider (midwife, nurse, PA, medical doctor)? Yes / No/unknown	
45	Was the delivery done at health facility or in the community?	

**Maternal Death investigation and reporting form for Health facility and community**		
This form MUST be filled by the professional health worker ***to investigate*** **death of a woman while pregnant or within 42 days of the delivery or termination of the pregnancy, irrespective of the duration and site of the pregnancy, from any cause related to or aggravated by the pregnancy or its management but not from accidental or incidental causes. This will be done** to identify and report primary cause(s) or key factors associated with the death with the view to take actions to prevent future maternal death.		
**Questions / Variables**		**Answers**
	Did she receive any herbal treatment?	
46	Was the deceased referred to any health facility or hospital? (Yes/No/unknown)	
47	If yes, how long did it take to get to the health facility? (hours)	
48	How long did it take for the deceased to receive any medical care or obstetrical/surgical interventions? (Hours)	
49	Specify the treatment and intervention received* **(see below)**<br>I.V. Fluids; Plasma; Blood Transfusion; Antibiotics; uterotonics (oxytocin, ergometrine, misoprostol); Anti-seizure drugs (Magnesium Sulphate); calcium Gluconate ;Oxygen; Anti-malarial; Other medical treatment; Surgery; Manual removal of placenta; Manual vacuum aspiration; Curettage, laparotomy, hysterectomy, instrumental delivery (Vacuum/forceps), corticosteroid, ARVs, Anti-shock Garment, Anti- hypertensive, Caesarian section, anesthesia (general, spinal, epidural , local) etc	
50	Primary cause of the Maternal Death	
51	Secondary cause of the Maternal Death	
52	Care provider title/qualification<br>Title:<br><br>Qualification:	
53	Was the care provider trained in Maternal care protocol or EmONC: Yes or No/unknown	
54	Provide analysis and Interpretation of the information collected (investigator's opinion including community view on this death):	
55	Maternal death notification date (day/month/year) the month should be written in words for consistency at all levels	
56	Maternal death investigation date (day/month/year) the month should be written in words for consistency at all levels	
57	Time (Hour) of death?	
58	How could this death have been prevented?	
59	Investigator (s) (Name, title/qualification)<br><br>(1)Name: Title: Qualification: Signature & Contact	

Maternal Death investigation and reporting form for Health facility and community		
This form MUST be filled by the professional health worker ***to investigate*** **death of a woman while pregnant or within 42 days of the delivery or termination of the pregnancy, irrespective of the duration and site of the pregnancy, from any cause related to or aggravated by the pregnancy or its management but not from accidental or incidental causes. This will be done** to identify and report primary cause(s) or key factors associated with the death with the view to take actions to prevent future maternal death.		
**Questions / Variables**		**Answers**
	(2)Name: Title: Qualification:<br>(3)Name: Title: Qualification:<br>(4)Name: Title: Qualification:<br>(5)Name: Title: Qualification:<br>(6)Name: Title: Qualification:<br>(7)Name: Title: Qualification:<br>(8)Name: Title: Qualification:<br>(9)Name: Title: Qualification:	

II. Neonatal Deaths investigation and reporting forms for Health Facility and Community

**Background**
• Globally, the number of deaths in children under five years of age has dropped significantly, from nearly 12 million in 1990 to about 6.3 million in 2013. (www.who.int/.../**child**.../**mortality**/**mortality**)<br>• Asphyxia, sepsis, preterm birth are major causes of Newborn deaths in Liberia. These major causes and other causes of Newborn deaths can all be prevented through cost effective, high impact interventions<br>• Unfortunately, globally, the proportion of child deaths occurring in the neonatal period has increased with neonatal deaths accounting for approximately 44% in 2012 of all child deaths in many countries.<br>• Though Liberia is among countries that have achieved MDG4, the proportion of under five deaths occurring in the neonatal period in 2013 was estimated at 26%.<br>• Greater investment and attention to the Newborn period, including the prevention of preterm births, stillbirths and the scale up of effective, low cost interventions such as antenatal corticosteroids, cord care and kangaroo mother care are needed to improve neonatal survival.**No index entries found.**
• **Surveillance Goal**
• **The overall goal of the MNDSR protocol is to guide an effective implementation and scale up of MNDSR in a systematic, standardized and integrated manner.**
• **Surveillance Objectives**
• Estimate and monitor neonatal mortality rates, including stillbirth rates.<br>• Identify risk factors for neonatal mortality to inform program decisions.<br>• Investigate all neonatal deaths including still birth in facilities and communities and take necessary action
**Standard case definition**
The death of a baby within the first 28 days of life.,(inclusive of the first day and first week of life which are the periods of greatest risk of death) including still birth
**Analyze and interpret data**
**Time:** Graph cases to construct weekly, monthly and annual epidemic curves<br><br>**Place:** Plot the location of cases and analyze the distribution.<br><br>**Person:** Count monthly cases and deaths. Analyze each case of neonatal death by cord care practices and KMC.
**Recommended public health action**
A case of neonatal death is a trigger for action at all levels (communities, health facilities, districts counties, and national).<br>Monitor trends and respond to each alert<br>All health care providers (professional and non-professional) should be trained on these protocols.

**MOH-Neonatal Death investigation and reporting form for Health facility and community**		
This form MUST be filled by the professional health worker ***to investigate*** **death** of a baby within the first 28 days of life (inclusive of the first day and first week of life which are the periods of greatest risk of death) ***including stillbirths.*** **This will be done** to identify and report primary cause or key factors associated with the death with the view to take actions to prevent future neonatal death.		
**Questions / Variables**		**Answers**
1	County	
2	District	
3	Name of health facility Reporting death	
4	Health facility catchment population?	
5	Place of death (facility or community)	
6	Name of the death place (Community/street/quarter, clinic, health center, district hospital, referral hospital or private hospital, on the way to health facility)	
7	Date of neonatal death (day/month/year) the month should be written in words for consistency at all levels	
**Record's unique identifier (year-Country code-District-site-neonatal death rank)**		
8	Age (in days) of the deceased	
9	Time of death (specify, during delivery, early neonatal period (1st 7 days) late neonatal period 8-28 days")	
10	Risk factor of the mother associated with the death	
	Did she have Fever? (Yes /No/ Unknown)	
11	Did she have Hypertension? (Yes /No/ unknown)	
12	Did she have Anaemia ? (Yes /No/ unknown)	
13	Did she have Abnormal Lie? (Yes /No/ unknown)	
14	Did she have diabetes? (Yes /No/ unknown)	
15	If still birth; SB – MACERATED OR FRESH?	
16	Was the delivery at health facility or in the community?	
17	Did she have convulsion/jerking (Yes /No/ unknown)	
18	Was there any bleeding (Yes /No/ unknown)	
19	Did she have premature labor? (Yes /No/ unknown)	
20	Did she have premature rupture of the membrane (PROM)? (Yes /No/ unknown)	
21	Did she have multiple births? (Yes /No/ unknown)	
22	Any other risk factor? Specify:	

MOH-Neonatal Death investigation and reporting form for Health facility and community		
This form MUST be filled by the professional health worker ***to investigate* death** of a baby within the first 28 days of life (inclusive of the first day and first week of life which are the periods of greatest risk of death) ***including stillbirths.* This will be done** to identify and report primary cause or key factors associated with the death with the view to take actions to prevent future neonatal death.		
**Questions / Variables**		**Answers**
23	**Postpartum/neonatal information**	
24	How long (hours) was the duration of labor	
25	Was the labor monitored by pathograph? Yes / No/unknown	
26	What type of delivery was it? (choose one from "1=spontaneous Vaginal delivery/normal delivery, 2=vaginal-assisted delivery (Vacuum/forcep), or 3=Caesarean section"	
27	Risk factor for the newborn	
28	Was the baby born asphyxiated? (Yes /No/ unknown)	
29	Was the baby born preterm? (Yes /No/ unknown)	
30	Was the baby born small for gestational age? (Yes /No/ unknown)	
31	Did the baby have any problem with temperature? (hypo/hyperthermia) (Yes /No/ unknown)	
32	Was the baby presenting with any or all of the danger signs? Yes or No. (jaundice, convulsion, chest in drawing, unable to suck/feed, difficulty breathing, no movement, infected cord)	
33	Was the birth assisted by a skilled care provider (midwife, nurse, PA, medical doctor)? Yes / No/unknown	
34	Was the delivery at health facility or in the community?	
35	Was the deceased referred to any health facility? (Yes/No/un known)	
36	If yes, how long did it take to get to the health facility? (hours)	
37	How long did it take for the decease to receive any medical care or surgical interventions? (Hours)	
38	Specify the treatment received*** (see below)**	
39	Primary cause of the Neonatal Death including still birth	
40	Secondary cause of the Neonatal Death including still birth	
41	Care provider title/qualification<br>Title:<br>Qualification:	

**MOH-Neonatal Death investigation and reporting form for Health facility and community**		
This form MUST be filled by the professional health worker ***to investigate*** **death** of a baby within the first 28 days of life (inclusive of the first day and first week of life which are the periods of greatest risk of death) ***including stillbirths.*** **This will be done** to identify and report primary cause or key factors associated with the death with the view to take actions to prevent future neonatal death.		
**Questions / Variables**		**Answers**
42	Was the care provider trained in newborn care protocol or EmONC? Yes or No/unknown	
43	Provide analysis and Interpretation of the information collected (investigator's opinion on this death)	
44	Neonatal death notification date (day/month/year) the month should be written in words for consistency at all levels	
45	Neonatal death investigation date (day/month/year) the month should be written in words for consistency at all levels	
	*** Specify:** Treatment/management received	
46	I.V. Fluids; Plasma; Blood Transfusion; Antibiotics; Anti-seizure drugs; Oxygen; Anti-malarial; Other medical treatment; Surgery; chlorhexidine, resuscitation, KMC, vitamin K, photo therapy, ARVs	
47	Investigator (s) (Name, title/qualification)<br><br>(1)Name: Title: Qualification: Signature & Contact<br>(2)Name: Title: Qualification:<br>(3)Name: Title: Qualification:<br>(4)Name: Title: Qualification:<br>(5)Name: Title: Qualification:<br>(6)Name: Title: Qualification:<br>(7)Name: Title: Qualification:<br>(8)Name: Title: Qualification:<br>(9)Name: Title: Qualification:	

MOH-Neonatal Death investigation and reporting form for Health facility and community	
This form MUST be filled by the professional health worker ***to investigate*** **death** of a baby within the first 28 days of life (inclusive of the first day and first week of life which are the periods of greatest risk of death) ***including stillbirths.*** **This will be done** to identify and report primary cause or key factors associated with the death with the view to take actions to prevent future neonatal death.	
**Questions / Variables**	**Answers**

Lee H, *et al. BMJ Open Qual* 2023; 12:e002147. doi: 10.1136/bmjoq-2022-002147

MATERNAL AND NEONATAL MORTALITY SUVEILLANCE CORE FUCNTIONS AND ACTIVITIES BY HEALTH SYSTEM LEVEL ACTIVITIES

Health System level	Identify	Report	Investigate	Analyze and Interpret	Responsible Person or Organization/Partner
Community	• Use simple case definition on maternal and neonatal deaths	• Report all deaths within 48 hrs to the nearest health facility;<br>• Report essential information on maternal and neonatal deaths including still births	• Support investigation activities<br><br>• Undertake verbal autopsy on causes of deaths	• Involve local leaders in observing, describing and interpreting the pattern and trends of deaths in the community;<br><br>Take appropriate actions	• CHVs<br>• CHDC<br>• District chairperson<br>• Representative women group<br>• Traditional healer<br>• Spiritual healer<br>• Town chief
Health facility	• Use standard case definition on maternal and neonatal deaths	• Report all deaths within 24 hrs. to the CHT<br>• Fill maternal and newborn death investigation and reporting form for immediate notification	• Head of Health facility investigate reported maternal and newborn deaths in the community and the health facility.<br>• County should provide logistical support to health facility	• Prepare and periodically update graphs, tables and charts to describe time, person and place<br>• A single case of maternal and neonatal death is an alert/emergency<br>• Interpret results<br>• Take appropriate action	• OIC& DHO<br><br>• RH supervisor<br>• MCH supervisor<br>• Surveillance focal person<br>• Second screener<br>• Skill CHV supervisor<br>• DHO

Lee H, et al. BMJ Open Qual 2023; 12:e002147. doi: 10.1136/bmjoq-2022-002147

Health System level	Identify	Report	Investigate	Analyze and Interpret	Responsible Person or Organization/Partner
County level	• Verify maternal and new born death at community and health facility levels using standard case definition<br>• Ensure reliable supply of data collection and reporting tools are available in all health facilities	• Report all maternal and newborn deaths to the national immediately<br>• Provide feedback to health facilities<br>• Improve communication at community and facility levels	• CHO and members of the committee Participate in investigations of maternal and newborn deaths at facility and community levels<br>• Review maternal and new born deaths report/records<br>• Provide (logistical) support and budget for the investigation (a word is missing)	• Verify to ensure the accuracy of all data<br>• Aggregate data from health facilities<br>• Monitor the trend of maternal and new born deaths<br>• Present results in tables, graphs and charts<br>• Periodically update graphs, tables and charts to describe maternal and neonatal deaths<br>• Calculate maternal and neonatal mortality rates (Please Insert Numerator and Denominator)<br>• Take appropriate actions	• Medical director<br>• Nursing Director<br>• County RH supervisor<br>• CHO,<br>• County Diagnostic supervisor<br>• County Surveillance officer<br>• Nurse Anaesthetist<br>• PA supervisor<br>• CHDD<br>• Clinical Supervisor<br>• RH<br>• County health promotion focal person<br>• County development Superintendent<br>• All health partners,

Health System level	Identify	Report	Investigate	Analyze and Interpret	Responsible Person or Organization/Partner
National level	• Define, update and ensure compliance with national policies and guidelines<br>• Set policies and procedures for reporting maternal and neonatal deaths	• Report and immediately notify appropriate authorities<br>• Provide feed back to County	• Ensure guidelines, SOPs for investigation are available<br>• Coordinate and collaborate with partners during investigation<br>• Provide (logistical) support and budget for the investigation	**Take appropriate action**	• MOH,<br>• National MNCAH Task Force and other line Ministries<br>• House standing committee on health<br>• Maternal and child health good will ambassador

Core Functions

Health System level	Prepare	Respond	Communicate	Evaluate
Community	• Participate in risk mapping on maternal and neonatal deaths<br>• Conduct community based surveillance on maternal and neonatal deaths	• Participate in response activities including home based care<br>• Mobilize local resources appropriate to reduce maternal and neonatal deaths<br>• Conduct immediate meeting to discuss any death of mothers and babies at community level	• Give feedback to the community about reported maternal and neonatal deaths	• Monitor and evaluate the preventive measures of maternal and neonatal deaths
Health facility	• Conduct risk mapping on maternal and neonatal deaths<br>• Conduct training of the community on actions to reduce maternal and neonatal deaths	• Investigate all maternal and new born death in the Health facility within 24 hours<br>• Take relevant measures to reduce maternal and neonatal deaths	• Give feedback to the community about reported maternal and neonatal deaths	• Assess community participation<br>• Monitor and evaluate the preventive measures of maternal and neonatal deaths
County level	• Conduct training for health facility staff<br>• Conduct risk mapping<br>• Support health facility based surveillance on maternal and neonatal deaths	• Mobilize resources to ensure sustainability of MNDSR activities<br>• Review maternal and new born death data immediately<br>• Take appropriate actions	• Give health facilities regular and periodic feedback on preventive measures<br>• Give feedback on surveillance and data quality findings	• Monitor and evaluate indicators for measuring quality of the surveillance system

Health System level	Prepare	Respond	Communicate	Evaluate
	• Coordinate the stakeholders in the planning and implementation of MNDSR interventions			• Conduct regular supervisory visits to health facilities
National level	• Set policies, procedures and training on reduction of maternal and neonatal deaths;<br>• Adapt guidelines (where appropriate) to reduce maternal and neonatal deaths;<br>• Develop messages for community education	• Mobilize resources to ensure sustainability of MNDSR activities at both national and county levels;<br>• Conduct periodic spot check monitoring and provide technical support to counties;<br>• Develop and disseminate bulletin on maternal and neonatal mortality	• Provide feedback to stakeholders on a quarterly basis;<br>• Review maternal and new born death data quarterly and recommend response actions	• Monitor surveillance indicators on maternal and neonatal deaths<br>• Conduct regular review meetings on maternal and neonatal deaths<br>• Conduct regular supervision
